# Genome Sequence of a Marine Alkane Degrader, Alcanivorax sp. Strain 97CO-6

**DOI:** 10.1128/genomeA.00087-18

**Published:** 2018-02-22

**Authors:** Xiaofei Yin, Xiao Luan, Angela Xu, Qian Li, Zhisong Cui, David L. Valentine

**Affiliations:** a Key Laboratory for Marine Bioactive Substances and Modern Analytical Technology, First Institute of Oceanography, State Oceanic Administration of China, Qingdao, China; b State Key Laboratory of Environmental Aquatic Chemistry, Research Center for Eco-Environmental Sciences, Chinese Academy of Sciences, Beijing, China; c College of Creative Studies, Biology, University of California, Santa Barbara, Santa Barbara, California, USA; d Department of Earth Science and Marine Science Institute, University of California, Santa Barbara, Santa Barbara, California, USA

## Abstract

Alcanivorax sp. strain 97CO-6 was isolated from a crude oil-consuming bacterial consortium, enriched from Yellow Sea sediments from China. Here, we present the draft genome of strain 97CO-6, which contains 3,253,423 bp, with a G+C content of 54.53%, as well as 2,931 protein-coding genes and 42 tRNAs.

## GENOME ANNOUNCEMENT

Alcanivorax is a cosmopolitan genus comprised of marine obligate hydrocarbonoclastic bacteria (OHCB), which play an important role in the biological cleanup of oil-contaminated marine environments (1–3). The competitive advantage of Alcanivorax species over other oil degraders is ascribed to the fact that its genome encodes a broad spectrum of systems for the catabolism of hydrocarbons (4). Alcanivorax sp. strain 97CO-6 (China General Microbiological Culture Collection Center accession number 3736) was isolated from sediments of the Yellow Sea (latitude 36.67, longitude 121.99, depth 17.8 m); this strain utilizes various *n*-alkanes as sole carbon and energy sources and produces a biosurfactant to enhance the bioavailability of the alkanes (5). The genome sequence of Alcanivorax sp. 97CO-6 could further inform our understanding of the remarkable catabolic capacity of alkane-degrading specialists in the Alcanivorax lineage.

Whole-genome shotgun sequencing of the Alcanivorax sp. 97CO-6 genome was done using Solexa paired-end sequencing technology by Shanghai Majorbio Bio-Pharm Technology Co., Ltd. (Shanghai, China). A total of 13,445,638 paired-end reads (180-bp and 800-bp libraries) were generated to reach a 398-fold depth of coverage using the Illumina HiSeq 2000 platform (Illumina, Inc., San Diego, CA, USA). The reads were assembled using SOAPdenovo version 1.05 (6, 7). The resulting genome sequence of Alcanivorax sp. 97CO-6 comprises 43 contigs (*N*_90_ = 78,739), with 3,253,423 bp and an average G+C content of 54.53%. Gene annotation was carried out using the NCBI Prokaryotic Genome Annotation Pipeline (PGAP) (https://www.ncbi.nlm.nih.gov/genomes/static/Pipeline.html), which was followed by manual editing. The annotation of the genome includes 2,931 candidate protein-encoding genes (with an average size of 960 bp), yielding a coding intensity of 86.50%. A total of 2,118 proteins could be assigned to clusters of orthologous (COG) groups; 42 tRNAs for all 20 amino acids and one 16S-23S-5S rRNA operon were identified using tRNAscan version 1.23 (8) and rRNAmmer version 1.2 (9), respectively.

Genome analysis of strain 97CO-6 revealed the existence of genes potentially involved in alkane degradation. Three genes encoding alkane 1-monooxygenase and two genes encoding cytochrome P450 enzymes were annotated in the genome of this strain. In addition, one gene encoding the haloalkane dehalogenase was found in the genome sequence.

The biosurfactant produced by strain 97CO-6 enhances the bioavailability of alkanes, and, correspondingly, genes potentially involved in biosurfactant production were found in the genome annotation. Two genes encode glycosyltransferases, which possibly provide the sugar moiety of the glucolipids and yield glucose lipid surfactants. The other two genes encode OmpA proteins, which are the active constituents of the biosurfactant alasan. Moreover, one gene encodes OmpW and the other encodes OmpH, both of which are possibly involved in emulsifier production. In conclusion, all of the genes related to both alkane degradation and biosurfactant production contain the necessary machinery for the adaptation of Alcanivorax sp. 97CO-6 in hydrocarbon-contaminated environments.

### Accession number(s).

This whole-genome shotgun project has been deposited at DDBJ/ENA/GenBank under the accession number PIXQ00000000. The version described in this paper is the first version, PIXQ01000000.
